# Assessing essential service provision for prevention and management of violence against women in a remote indigenous community in Amantaní, Peru

**DOI:** 10.1186/s12939-023-02012-3

**Published:** 2023-10-03

**Authors:** Maria Calderon, Carla Cortez-Vergara, Laura Brown, Hattie Lowe, Blenda Abarca, Marta Rondon, Jenevieve Mannell

**Affiliations:** 1 HAMPI: Consultores en Salud, Lima, Peru; 2 Universidad Peruana Cayetano Heredia, Lima, Peru; 3 Institute for Global Health, University College of London, London, UK; 4 Instituto Nacional Materno Perinatal, Lima, Peru

**Keywords:** Violence against women, Indigenous people, Emergency services, Mental health services, Health services research, Peru, Latin America, Indigenous populations, Indigenous health services, Qualitative research

## Abstract

**Background:**

Women living in indigenous communities in Peru currently experience extremely high rates of intimate partner violence (IPV). Over the past 10 years, there has been a large multi-sectoral initiative to establish a national network of *Centros de Emergencia de la Mujer* (Women’s Emergency Centres) that integrate health and police services, and substantial increase in efforts from non-governmental organisations in supporting survivors of violence. However, there is currently little evidence on how existing services meet the needs of indigenous women experiencing violence in Peru.

**Methods:**

As part of a broader mixed-methods participatory VAWG prevention study, we assessed existing service provision for women experiencing violence in an indigenous Quechua community from Amantaní, Peru. This involved 17 key informant interviews with legal, government, police, and civil society representatives. We used the UN Women Essential Services Package for Women and Girls Subject to Violence framework to guide our analysis.

**Results:**

Participants identified major gaps in existing services for indigenous women survivors of violence in Peru. They discussed survivors and perpetrators not being identified by the health system, a lack of IPV response training for health professionals, IPV not being prioritised as a health concern, and a lack of health services that are culturally appropriate for indigenous populations. Survivors who report to police are often treated poorly and discriminated against. Legal systems were perceived as insufficient and ineffective, with inadequate legal measures for perpetrators. While legal and policy frameworks exist, they are often not applied in practice. Service provision in this region needs to adopt an intercultural, rights based, gendered approach to IPV response and prevention, considering cultural and linguistic relevance for indigenous populations.

**Conclusion:**

The role of structural violence in perpetuating indigenous women’s experiences of violence and undermining their access to services must be central to designing and implementing appropriate policies and services if they are to meet the needs of indigenous women in Peru.

## Key messages

- Intimate partner violence is a serious health and human rights issue affecting women who have been traditionally discriminated against and excluded from support services in the Andes

- Services available in the region need to adopt an intercultural, rights based and gendered approach to IPV response and prevention, considering cultural and linguistic relevance for indigenous populations in Peru

- Structural violence needs to be considered in assessing indigenous women’s experiences of violence and their access to services

## Introduction

Approximately one in three women globally experience intimate partner violence (IPV) in their lifetime, with consequences for their physical and mental health [1]. IPV, the most common type of violence against women and girls (VAWG), refers to “behaviour by an intimate partner or ex-partner that causes physical, sexual or psychological harm, including physical aggression, sexual coercion, psychological abuse and controlling behaviours” [1]. The 2019 Demographic and Family Health Survey in Peru estimated that 57.7% of ever-partnered women between the ages of 15 and 49 had been subject to some type of violence by an intimate partner in their lifetime [2]. While these figures are high on a global level, they also mask even higher prevalence rates across different groups and regions within Peru. The same survey estimated that 62.5% of women of native origin, including women self-identified as Quechua, have experienced some form of violence by an intimate partner in their lifetime. Deep-rooted structural factors perpetuate and normalise violence against women and girls (VAWG) in Peru [3, 4]. Indigenous women in Peru experience high rates of gender-based violence driven by legacies of colonialism, systemic inequality and discrimination [5, 6]. Moreover, indigenous women are often disproportionately excluded from health, social and legal services for violence prevention due to socio-cultural, geographical and institutional barriers [6–10].

Peru’s history of patriarchy, ethnic, racial and religious discrimination, colonialism, and the relatively recent armed conflict (1980s-2000s) has shaped the higher rates of violence experienced by women and girls, particularly in indigenous communities. As in many other Latin American countries, guerrilla groups formed in the 80 s and 90 s in Peru. The conflict, including military response led to almost 69,280 deaths or disappearances [11, 12]. Most of the women who were victims were Quechua speaking, young, in the peasant class, and with low levels of education [11, 12]. Furthermore, 78% of families displaced by the conflict were headed by women but authorities only gave documentation to men to whom women were forced to marry to be recognised by society and have a legal identity [3]. People affected by trauma did not seek mental health support because there was none, either in the rural areas or in urban settings. The mental health system at the time consisted of some poorly resourced specialised psychiatry hospitals, which were only in Lima, and only attending chronic psychiatric conditions like schizophrenia [13]. Additionally, more than 200,000 poor women from indigenous communities or rural areas in the Andes and Peruvian Amazon were sterilised as part of a national programme in the 90 s. Those programs failed in part to lack of health care provider training [11, 12]. This extended to a lack of understanding of the process of informed consent, and controversial interpretations of whether the women had actually understood what was asked of them. Indeed, in about 20 cases, there was no informed consent form [14]. This history has contributed long-lasting negative impacts on community cohesion, as well as gender, power and family relations [15]. It has also contributed to an increased risk of anxiety, depression, substance abuse and impulsivity in younger generations [16].

To address the high rates of VAWG in the general population, the Peruvian government has established laws to prevent violence against women and facilitate the punishment of aggressors [17] (see Table 1). In 2020, the National Program for the Prevention and Eradication of Violence against Women and Members of the Family Group (AURORA) was created. This programme aims to design and implement actions and policies for the attention, prevention and support of people involved in acts of family and sexual violence at the national level [18]. There has been an intersectoral effort by the Peruvian Ministry of Health and the Ministry of Women and Vulnerable Populations to establish a network of *Centros de Emergencia de la Mujer* (CEM; Women’s Emergency Centres) [19] that aim to provide comprehensive psychological, medical and legal support free of charge for women experiencing violence. These services are distributed across the entire country and Puno, along with Ancash and Cusco, have the largest number. However,the Ombudsman has found that these are understaffed, they do not coordinate well with health and other services and there are serious problems with staff preparedness. Rurality, illiteracy and ethnic factors [would] render these services even more ineffective for women in Amantaní [20, 21].

**Table 1:** Principal laws and policies against VAWG in Peru (all still apply)

- Law 30364 (to prevent, punish and eradicate violence against women and members of the family group) which establishes mechanisms, measures and comprehensive policies for the prevention, care and protection of victims, as well as reparation for the damage caused within the measures within a maximum period of 72 h after the complaint. Includes the issuance of necessary protection measures and reference to the Criminal Prosecutor's Office to initiate the process. This law also states that, if an obvious crime is noted, the aggressor must be arrested immediately. Promulgated in 2015. (Congreso de la Republica del Peru [17])
- Law 30314 (to prevent and punish sexual harassment in public spaces) which sanctions acts, comments and insinuations of a sexual nature, obscene gestures, improper touching and exhibitionism. Promulgated in 2015. (Congreso de la Republica del Peru [17])
- Law 27942 (on the Prevention and Punishment of Sexual Harassment) which addresses sexual harassment in the work environment. Promulgated in 2003. (Congreso de la Republica del Peru [17])
- Law 29819 which incorporates feminicide as a crime in the Criminal Code with a punishment of deprivation of liberty [jail sentence] for not less than fifteen years. Promulgated in 2011. (Congreso de la Republica del Peru [17])
- National Program for the Prevention and Eradication of Violence against Women and Members of the Family Group – AURORA: This program aims to design and implement actions and policies for the attention, prevention and support of people involved in acts of family and sexual violence at the national level. This program is attached to the Ministry of Women and Vulnerable Populations. Created in 2001 (AURORA 2020 [18])
- Protocol for joint action between Women's Emergency Centers (CEM) and Health Centers for the care of victims of violence against women and members of the family group: This protocol defines criteria and procedures for the comprehensive and effective coordinated action of two services that are part of the comprehensive care circuit for victims of violence. Created in 2009 (Observatorio Nacional 2019 [19])

Given this background, the specific aims of our study were to: 1) assess the services available for women survivors of IPV living in a rural indigenous community in the Andes, and 2) assess the relevance of the UN Women tool for service provision for indigenous communities in Peru.

## Methods

### Study setting

The island of Amantaní is inhabited by a predominantly Quechua-speaking indigenous population. Located in Lake Titicaca in the Puno region of southern Peru, it has an estimated 4,255 inhabitants and an area of 9.28 km.^2^ in which agriculture and tourism are the main sources of income. At 3,854 m above sea level, the island is only accessible by boat, a journey which takes between two to four hours from the nearest towns. Amantaní is one of the poorest districts in Puno, with 93.8% of the population classed as living in poverty, and 55% classed as living in extreme poverty [22, 23]. Access to healthcare is limited with only one primary health care facility. If specialised care is required, patients are referred to the Hospital of Puno. While free medical care is accessible through the Integral Health Insurance (once registered in Puno), patients are required to cover the cost of transport to the hospital upon referral. A 2019 health needs assessment in Amantaní highlighted poor healthcare access and a lack of awareness of disease prevention [24]. While the exact prevalence of intimate partner violence in Amantaní is unknown, high prevalence rates in the surrounding region of Puno and among women of native origin suggest that women in Amantaní face high rates of violence in their intimate relationships [23].

### Research development and data collection

As the first phase of a mixed-methods, participatory research project on VAWG prevention in Amantaní (the EVE Project [25]), this study was designed as an initial service assessment as part of a broader research project. This assessment provided a means of ensuring that the EVE Project was relevant to the local context and following international ethical guidelines in VAWG research [26–28]. As women may experience distress and re-traumatisation while participating in VAWG research, or they may disclose that they are currently experiencing abuse [29], the WHO’s ethical guidelines suggest that research teams should be trained to refer women to appropriate local services. To adhere to this guideline, service assessments such as ours are particularly important in research with women from indigenous communities, where it may be more difficult to link research participants with local support services and tailored supports may be needed [30, 31].

Our team includes researchers based in London (United Kingdom), Lima and Puno (Peru) with backgrounds in global health, sociology, violence against women prevention and mental health. Between January and March 2021, we conducted 17 qualitative semi-structured interviews to explore conceptualisations of IPV, services available to women experiencing violence, policies/legal frameworks to address VAWG and theories around the drivers of the high prevalence of IPV in Peru. The interviews were conducted in Spanish by three Peruvian members of the research team via video-call due to COVID-19 travel restrictions. Interviews were recorded, transcribed and translated into English for collaborative analysis.

### Participant recruitment

Recruitment began in December 2020, during the COVID-19 pandemic. We approached potential participants by telephone, text messages, WhatsApp and e-mail. We used a combination of purposeful and snowball sampling to recruit key informants (KIs). We contacted representatives from key sectors (health, judicial or legal, protection, etc.) and presented the study objectives via video-call. We then consulted with these same representatives to identify key actors to be interviewed. Consent forms were given to all interested KIs who were asked to explicitly give their consent via e-mail or provide pictures of their signed consent forms. Informants from outside Puno were included in the study when they had extensive experience working with ​​victims of IPV or with indigenous populations (e.g., local experts, representatives of indigenous communities). Following the interviews, KIs were asked to recommend other suitable participants. Key informant details are presented in Table 2.

**Table 2:** Key informant main role descriptions

Judicial and police actors: KI-1, KI-16, KI-13, KI-14, KI-15	Representatives of the Prosecutor’s Office at national and regional level. The Prosecutor’s Office’s function is to promote ex officio or at the request of a party, action in defense of legality and public interests protected by law. They ensure the independence of the legal bodies and the correct administration of Justice. They represent society in legal proceedings
	Representatives of Judicial Power at the regional level. The Judicial Power is in charge of exercising and administering justice in the country in accordance with the Constitution and laws, guaranteeing the defense of the goods and rights of citizens
	Police representative in Puno
Health system actors: KI-3, KI-5, KI-8, KI-12, KI-16, KI-17, KI-6	Representatives of the Ministry of Health at the regional level, including regional coordinator of the Mental Health Control and Prevention Program
	Representatives of the Ministry of Women and Vulnerable Populations. This ministry is in charge of directing, coordinating and managing public policies for the prevention, care, punishment and eradication of gender-based violence
	Representatives of the Women’s Emergency Centers that are coordinated by the Ministry of Women and Vulnerable Populations
	Health professional responsible for the local primary care center
Advocates: KI-2, KI-4, KI-7, KI-9, KI-10, KI-11	Representative of the Ombudsman. The Ombudsman's Office is part of the Public Ministry, and exercises its functions under the supreme direction of the Attorney General of the Nation. It is responsible for ensuring the promotion, exercise and dissemination of Human Rights
	Members of the Catholic church that are involved in VAWG prevention and victim support initiatives
	Member of indigenous groups rights organisation
	Academic with national expertise in researching and responding to violence against women in Peru

*KI* Key Informant code

### Data analysis

Qualitative data were analysed thematically. We used the UN Women’s Essential Services Package for Women and Girls Subject to Violence [32] to assess the service provision for IPV prevention and management for the indigenous community of Amantaní. This framework includes four components: 1) principles underpinning delivery of all essential services, 2) common characteristics describing a range of activities common across all areas which support the effective functioning and delivery of services, 3) essential actions under health, justice and policing and social services, and 4) essential actions for coordination and governance of coordination (Table 3).

**Table 3:** Summary of key findings according to sector and recommendations for improving services for indigenous communities

UN Women Framework principles, common characteristics and foundational elements	UN Women Framework Essential services and actions	Key findings	Recommendations for improving services for indigenous communities
**Principles** 1. A rights-based approach 2. Advancing gender equality and women’s empowerment 3. Culturally and age appropriate and sensitive 4. Victim/survivor centred approach 5. Safety is paramount 6. Perpetrator accountability **Common characteristics** 1. Availability 2. Accessibility 3. Adaptability 4. Appropriateness 5. Prioritise safety 6. Informed consent and confidentiality 7. Data collection and information management 8. Effective communication 9. Linking with other sectors and agencies through referral and coordination **Foundational elements** 1. Comprehensive legislation and legal framework 2. Governance oversight and accountability 3. Resource financing 4. Training and workforce development 5. Gender sensitive policies and practice 6. Monitoring and evaluation	**Health**		**Accessibility** • Outreach mental and physical health services to remote communities **Appropriateness** • Service provision in local languages **Rights based approach** • Integrating indigenous governance structures and justice concepts **Culturally appropriate and sensitive** • Inclusive and non-discriminatory services • Intercultural competency training for service providers
	1. Identification of survivors of IPV 2. First line support 3. Care of injuries and urgent medical treatment 4. Sexual assault examination and care 5. Mental health assessment and care 6. Documentation (medico-legal)	VAWG survivors are not identified by the health system because of inadequate screening tools and protocols	
		Lack of VAWG training for health professionals including on supporting survivors, sexual assault examination and required documentation for trial purposes and mental health	
		VAWG is not perceived as a priority health concern leading to delays in service provision	
		Health services are not accessible for indigenous populations	
		Health services are not culturally appropriate for indigenous populations	
		Indigenous survivors of IPV face stigma and discrimination when accessing health services	
	**Justice and policing**		
	UN Women Framework components 1. Prevention 2. Initial contact 3. Assessment/investigation 4. Pre-trial processes 5. Trial processes 6. Perpetrator accountability and reparations 7. Post-trial processes 8. Safety and protection 9. Assistance and support 10. Communication and information 11. Just sector coordination	VAWG survivors who report to the police are treated poorly and often blamed for the violence they experience	
		Justice and policing systems are insufficient in rural areas because of underfunding	
		Limited resources result in slow trial processes which deter survivors from continuing with their case	
		The legal response to perpetrators is inadequate, they are not sufficiently punished which can lead to re-perpetration	
		Corruption in the justice system works in favour of perpetrators	
		Traditional legal systems and concepts of indigenous populations should be integrated into national VAWG response	
	**Social services**		
	1. Crisis information 2. Crisis counselling 3. Help lines 4. Safe accommodation 5. Material and financial aid 6. Creation, recovery, replacement of identity documents 7. Legal and rights information, advice and representation, including in plural legal systems 8. Psycho-social support and counselling 9. Women-centred support. Children’s services for any child affected by violence 10. Children’s services for any child affected by violence 11. Community information, education and community outreach 12. Assistance towards economic independence, recovery and autonomy	Available services are seen as ineffective and under resourced	
		Coordinated psychological, medical and legal services provided by Women’s emergency Centres but there is a lack of continuity of care	
		Economic barriers insufficiently addressed	
		Some positive indigenous-focussed initiatives although these are limited to economic empowerment	
		Services need to be tailored to indigenous values	
	**Coordination and governance of coordination**		
	National level: 1. Law and policy making 2. Appropriation and allocation of resources 3. Standard setting for establishment of local level coordinated responses 4. Inclusive approaches to coordinated responses 5. Facilitate capacity development of policy makers and other decision-makers on coordinated responses to VAWG 6. Monitoring and evaluation of coordination at national and local levels Local level: 1. Creation of formal structures for local coordination and governance of coordination 2. Implementation of coordination and governance of coordination	Law and policies are strong with national efforts towards coordinated responses	
		Moving towards decentralisation of services to improve access for women in rural areas	
		Legal and policy frameworks are not being applied consistently in practice	
		Lack of coordination between different sectors with contradictory approaches	
		Where regulatory and policy frameworks have been applied, these have not benefited indigenous communities, who are often outside of the formal health and social services sectors	

Five authors participated in a collaborative qualitative analysis. The analysis aimed to compare services in Peru to those proposed in the UN Women framework and identify gaps. As such, coding was done deductively based on the essential services framework using a coding matrix created in Microsoft Excel. We coded data as either evidence of a component of the framework, or lack of a component. Initially, five interviews were double coded to achieve consensus on the initial coding strategy. The remaining interviews were divided between the five authors who each coded between two and three transcripts. Discrepancies were dealt with through jointly analysing the interview until consensus was reached. Next, we grouped codes into themes. We also included inductive themes that related specifically to the unique needs of indigenous communities, which were not captured by the UN Women framework as this topic was consistently considered fundamental in all interviews. Themes were organised using matrices in Excel.

## Results

The overarching themes are presented below according to the four main components outlined in the UN Women framework: health, justice and policing, and social services sectors, and coordination and governance. Table 3 presents a summary of these findings. KIs coding used to reference quotes are presented in Table 2.

### Health

#### IPV survivors are missed by the health system

Most participants raised concerns about survivors not being identified by the health system. General health screening forms that included questions to identify survivors usually contained closed questions, which limited opportunities for women to disclose violence:

*The problem we have had, and we are improving this all the time, is that out of 10 screenings or 100 screenings, we only detect five or six women that have experienced violence. We know that this is not the case.… the form has closed-ended questions; Yes or No. If the answer was no, you made an X and you didn't ask any more information. [KI-3]*

Participants from a variety of sectors spoke about the limitations of the health system in paying specific attention to possible acts of violence when this was not seen as the reason for the consultation, particularly for women seeking emergency care services:

*But if women go to the hospital, they are not assessed as they should be. They do not move quickly [i.e. the case is not quickly assessed]. If a woman who has been assaulted goes [to a hospital], covered in blood, then maybe they will move and have everything checked. Nevertheless, she will be treated for that problem only [physical injuries], but it is not as it should be. [KI-7]*

Most participants highlighted how women from indigenous communities lack opportunities to report violence to the health sector because they don’t use health services in the first place. They explained this as a structural issue, whereby a lack of economic resources prevents women from being able to leave the community to attend health facilities located in the city:

*So, imagine a woman in our community that must go to the emergency centre in the province. She has to leave the community to go there… and what about the money? If you go there, you are going to leave your things behind, your children… and you have no money. Then, many times they say "It’s better that I endure this situation, right?... He brings me 10 soles to eat, I do my chacra [farming], in my house with my children.... I’m going to endure”. They have no other choice. Sometimes when women make a complaint, they withdraw it precisely because of this situation. [KI-2]*

*We have CEMs [women’s emergency centres] in rural areas, however only a few now […]. We are thinking to open more CEMs that are specific for indigenous populations (Quechua or Aymara)... the majority of the CEMs are in cities. [KI-17]*

Some participants raised concerns about how discrimination in the health system often deters survivors and their allies from reporting:

*If we approach them [emergency services providers] to make a complaint, they immediately start to make fun of the way we say things. [KI-10]*

#### Lack of VAWG training for health professionals

Several participants raised the issue of a lack of training available for health professionals on handling cases of violence. Soft skills training was recognised as necessary to providing better support for survivors:

*I feel that one of the shortcomings is the lack of staff training, the lack of awareness of the treatment that should be given to women who suffer physical or psychological violence and also those who have been raped, girls, boys or women. [KI-9]*

A few participants also raised specific concerns about a lack of training on sexual assault examinations and the required procedural documentation:

*I feel that what we need is training, but also mechanisms, for example, to ensure that a girl who has been sexually assaulted can continue to keep the evidence that the judge requests, or how to ensure that the health system keeps a proper record of what was found. [KI-9]*

When asked about the needs of indigenous communities, several participants across sectors raised concerns about an absence of mental health specialists in indigenous and rural communities, another gap in training for health providers. General practitioners and sexual and reproductive health practitioners do not have integrated mental health training. There is also limited awareness of the newly implemented community-based mental health network (Centros de Salud mental Comunitaria); practitioners think only of the psychiatric hospital in responding to the emotional needs of survivors of IPV [33]:

*There is no mental health hospital here in Puno. It is very disorganised. On one occasion I received a woman here in the house […] but her behaviour led me to look here in Puno if there was a [mental health] specialist […] her behaviour scared me, she was attacking other people who were here [in the refuge]. I wanted to take her out of the refuge immediately, but there are no specialists. So, they [health centre] said “We'd better send her to Lima. Yes, there is this psychiatrist in Lima”. [KI-7]*

In addition, some participants mentioned that there are no health services provided in Quechua or Aymara close to Amantaní.

The absence of appropriate training was perceived as having significant consequences for addressing IPV. For example, some participants discussed how limited training can lead to potential perpetrators not being recognised until it is too late:

*One of the teachers, who was a bit older, stood up and said to me: "I know this young man [the aggressor], because he was my student […] he was an introverted person, he didn't have many friends, but he always reacted violently." The question is, did anyone intervene...? I understand that he was also in the military. So, there were several filters, and we never screened this person. We never noticed this kind of behaviour because we thought it was normal. [KI-3]*

#### IPV is not a priority health concern

Many participants discussed a disregard for survivors of violence by the health system, largely because healthcare providers didn’t perceive violence as a priority. They gave examples of significant delays in service provision and a lack of empathy for survivors:

*We waited 3 hours and I could see that the woman was suffering, and I felt sorry for her. So, I said "When!?" and they told us "Maybe the doctor will not come, you better come back tomorrow." [KI-7]*

One participant raised concerns about health services staff resisting reporting violence without physical evidence and difficulties in assessing and documenting psychological damage:

*They ask for proof, evidence. The only thing they can give evidence of is physical, isn't it? Proof of physical violence. But they [perpetrators] say: “You are good for nothing, you are ignorant, you don't know how to raise your daughter, you are a bad mother” and so many other things they say to her, how can there be proof? [KI10]*

Another participant believed the disregard for violence within the health system came from the need to address IPV elsewhere, as part of a comprehensive strategy for prevention rather than response. Participants highlighted drivers of IPV, such as alcohol consumption, as needing to be targeted:

*In terms of primary prevention, there is little that the Ministry of Health or health personnel can do. You can participate in community efforts to build a culture of peaceful conflict resolution and contribute to reducing gender inequality through how the health facility deals with women and how you teach women what their rights are and how they need to see them respected, but there is little they can do in terms of violence prevention[...] You can reduce the use of alcohol, that is something that health services can do directly and that would contribute to reducing about 34% of the violence related to alcohol use. [KI-4].*

### Justice and policing

#### Women survivors who report to police are treated poorly

Most participants shared specific examples of how survivors are treated poorly by police officers:

*We know that there is still a lack of preparation [by the police] to receive the [victim’s] complaint. I understand that there must be some protocol, but what is lacking is how they treat people. There is no empathy. [KI-3]*

Police inaction in response to IPV was seen as problematic and leading to mistrust. As one participant discussed, women are often blamed for the violence they experience:

*She goes to the police station, and they say: "It's your husband. Surely you have behaved badly, it is not someone else who is beating you, it is your husband". [KI-10]*

Maltreatment was attributed to police not wanting to work on violence cases:

*It is true that for the police when they are assigned to violence against women, violence against the family, it is like a kind of punishment, they take it badly and then they take it out on the victims. [KI-4]*

This is particularly relevant for indigenous communities where police services are often the only local service available for women experiencing violence:

*There may be institutions that do not exist in such remote places, there is no prosecutor's office, there are no courts or others […] However, there is a police office, you will always find a policeman around in any place. [KI-1]*

#### The legal system is insufficient and ineffective

Many participants said justice and policing systems are inefficient, especially in rural areas, and described this as a result of underfunding. Participants felt that recent legislative changes related to IPV, have not been supported by adequate resources for implementation:

*The negative side is that there has not been an increase in the number of people who deal with these complaints, because the law did not come with a budget to increase the number of police and to increase the number of prosecutors, so each one has a backlog of complaints to handle, and therefore once you have made the complaint to get the prosecutor to do the investigation and pass it to the judge to get the protective measures that theoretically should come within 48 hours according to the law, it takes six months. [KI-4]*

Legal representatives described how limited resources contribute to inadequate trial processes, which are slow and deter survivors from continuing with their cases:

*There is also a great weakness of a slowness in the judicial processes, a slowness in the investigations, a slowness from when the woman makes the complaint at the police station and that this report goes to the forensic doctor and then goes to the court or to the public ministry so that later there are, at least, the protection measures, a lot of time passes and the woman gets tired, she gets bored. [KI-9]*

A proposed solution to address the weaknesses of the legal system in persecuting IPV within indigenous communities was to increase community autonomy, and integrate local responses into the national IPV response:

*We are just in the process of constructing the proposal to modify the statutes that we have been working on. Because autonomy must also be respected. That is why we talk about special justice and ordinary justice, but this special justice is totally abandoned, this special justice is not strengthened. We have the ordinary justice, the corrupt, racist ordinary justice we say, because everything is in Spanish, everything is seen from the cosmovision of cities, not necessarily the indigenous thought… then all this limits you. What we want is for the special justice to be strengthened. The community members themselves, in an assembly, decide if they have to sentence the person and then they coordinate with the ordinary justice. [KI-2]*

Efforts to incorporate traditional laws were also mentioned by legal representatives:

*…in peasant communities, you have to respect multiculturalism, customs. These customs must not be totally eradicated because the Constitution says that the customs of ancestral communities must be respected. We need to adapt those, make them understand that there are certain behaviours, which, although for them it is very natural, it damages the physical and psychological integrity of people. Because we are not going to be stuck in the 19th century with negative customs, rather you have to assimilate them, understand them and respect the customs that are positive and ancestral [KI-1]*

#### Inadequate legal measures for perpetrators

Overall, participants raised concerns around the legal system’s response to perpetrators and how they often fall through cracks in the system. Most participants discussed inadequate punishment of perpetrators by the courts:

*There is no justice in this country. Moreover, they say “let's put all machistas [sexists] and rapists in jail” […] and then the jails are full…And what do they do there [with the perpetrators]? They come out threatening, and tougher. What kind of justice do we have in our country? [KI-2]*

Other participants described punishments as not fitting the severity of the crime, highlighting how this encourages further acts of violence:

*“That's not a crime, it's an offence” they [police, judiciary system] say. And who would believe it?... Out of 100, One becomes a crime, 99 don’t. So, in this situation the man comes out from the police feeling strengthened...he says, "nothing is going to happen to me" and he goes back home. [KI-4]*

Corruption in the justice system also prevented men from being punished appropriately for IPV:

*Violence is not going to go away easily. One, because the authorities that administer justice are too slow and are easy to corrupt. That's the problem. If a man is reported and goes to the police and offers him money or something else, what do you think? The evaluation and the preliminary report come back mild, and the guy walks free…[KI-12]*

### Social services

#### Mixed perceptions about the effectiveness of available services

The majority of participants discussed the implementation and wide dissemination of an emergency national helpline for survivors of violence by the Ministry of Women and Vulnerable Populations. However, there were concerns about its ineffectiveness:

*Other people say "No, it's just a joke, it's like the 105 call to the police and they never answer”. [KI-12]*

In addition to the helpline, the government has implemented Women’s Emergency Centres (Centros de Emergencia Mujer, CEMs) to provide a coordinated VAWG response by psychological, medical and legal services. However, participants who had engaged with CEMs felt that continuity of care was not delivered effectively due to limited resources:

*I see that the service provided by the CEM with a social worker, for example, at least it helps, the psychologist also [helps] when I ask for help, but as I said, I understand that the services are overloaded with work, but sometimes it takes a lot of time. I would like that when the woman arrives, there is continuity in care. But there is not. They tell them "I'll be here at such an hour", but the person does not arrive. The next day the woman is still waiting. [KI-7]*

Another reason given for the CEM’s ineffectiveness is its failure to address the economic realities for women who leave violent relationships:

*They provide services with this work, professionals like psychologists or social workers and the police station; they work coordinating to support the woman. But I feel that it is not enough, because when women leave their homes, when they have suffered violence, they leave without clothes, without money, right? [KI-7]*

#### Creating services tailored for indigenous populations

A small number of participants discussed the actions of organisations trying to provide financial support for indigenous survivors of violence through business creation initiatives. One such initiative is working directly with indigenous populations living in Puno city:

*This house has also been lent to them while the woman was working and raising money, so she has already raised a small amount of capital, and can now open her own place, for example, a cosmetology place or a restaurant. We have helped in this way. Maybe we do not solve anything, but at least we empower and promote the fact that women know how to do things. [KI-10]*

Beyond addressing economic barriers to IPV service access, many participants also raised the need for service provision to be specifically tailored to indigenous values:

*There has to be theoretical work in the language vernacular that allows us to understand that there are some universal values, for example, treating each other with affection, feeding everyone equally… which are values that you also see in the communities that speak Aymara and Quechua […] I think that we have to find these values and these truths and they are universal so that it doesn't seem that we are simply trying to impose some culture and destroy the culture that existed, which is also done a little bit, but we shouldn't do that. [KI-4]*

### Coordination and governance

#### Strong laws and policies with national efforts towards coordinated responses

Several participants discussed how relevant policies are in place (see Table 1) and that improvements are being made on a regular basis.

As one participant described:

*Law 30364, which is the law against violence against women and family members, is a law that has been revised and improved countless times. The first time it came out it was dreadful and over the 20 years it has improved a lot and we can say that it is getting much closer to international recommendations. [KI-4]*

Participants also mentioned efforts by the government to decentralise services and improve access for women in rural areas:

*Last year, 2020, in June, the new operations manual of the Aurora National Program was approved, and among the new features, territorial units were created. We need to consider that before its creation, under the National Program against violence, it had a working structure where all its units were in Lima and the services were decentralised at the national level but monitored from Lima. [KI-7]*

#### Legal and policy frameworks not applied in practice

Despite the presence of legal frameworks and policies at the national level, participants highlighted a lack of coordination between sectors:

*At the institutional level we implement well, but slowly, as if something is wrong. We are not efficient […] It is like we take [too much] time, and it is like we are also discouraging the assaulted woman and the aggressor, going from office to office and that could be done in a short period of time. She does not need to go for hours, until 11 o'clock at night without lunch. So, things like that are discouraging. I do not know if it is a matter of organisation, but I find that very difficult. [KI-7]*

They also highlighted contradictory approaches to IPV across sectors and the need to adapt these approaches to the multiple contexts in the country, including considering the language used in written protocols across sectors:

*The point of the Ministry of Health, there is a problem with the protocols. If you compare, for example, the protocol for joint care of the women's emergency centres and health facilities that was written by the Ministry of Women's Affairs and you compare the language of the clinical practice guidelines for attention to violence, now they are writing a new one but the one from the Ministry of Health, you realise that the one from the Ministry of Women's Affairs has a more modern approach and is better adapted to the recommendations of the WHO on violence against women. They insist on the theory that, well, these are the global recommendations, but we have to adapt to the national reality and the first fallacy is that we don't know what the national reality is because […] the research that is done here is of very poor quality when it is done. [KI-4]*

Participants described progress in the implementation of regulatory frameworks and decentralisation of the VAWG response from Lima to other regions doing little to address the needs of indigenous communities, which are often outside of the formal health and social services sectors. An indigenous rights activist highlighted the need for reflection of privilege by those within the system as a means to provide fair justice:

*Of course, those who are in the services must enter a process of reflection, a process of questioning their privileges as well. The need to do real justice because it does not exist. I am sure they have been given a talk, but with a talk you do not change anything. Sometimes this process is permanent, the justice worker must be in a permanent process of reflection... Because when you go to make a denunciation, they tell you "Ah, you… what have you done now?" [KI-2]*

## Discussion

Our results show how services for indigenous women survivors of IPV in Amantaní have many shortcomings, despite an appropriate legal framework and the existence of national and international guidelines and protocols. Implementations of these are scarce due to the extreme lack of funding in this area. In the health sector, survivors are missed, training for personnel is inadequate and IPV is not a priority health concern. In the justice and policing sector, survivors who report to police are treated poorly, the legal system is inadequate and ineffective, and legal measures for perpetrators are insufficient. There are mixed perceptions about the effectiveness of social services, and despite efforts to create services tailored for indigenous populations, they are often inaccessible or unknown by the target population.

Indigenous women who experience IPV face numerous challenges in accessing available services in Peru, which could be partly due to pre-existing structural inequalities and discrimination [34, 35]. Our findings highlight a notable example: the absence of service provision in Quechua or Aymara – the mother-tongues of most women in the Puno region – and a lack of intercultural competency by service providers. This is in line with the International Working Group for Indigenous Affairs and Work International Organization Report, which found that a large proportion of indigenous women in Peru (60%) perceived the discrimination they experienced in the public services and daily life to be due to their indigenous identity [36]. Furthermore, as Amantaní is a remote island in the highlands of the Peruvian Andes, many indigenous women are unlikely to afford the cost of travel to the mainland to report violence, creating another critical barrier to accessing services. Importantly, within the island, medical care is provided by a general practitioner from the rural services program (which is mandatory for new graduates seeking to apply for medical specialty training). This position has a yearly rotation, thereby preventing continued engagement with the community, and it has been criticised for a lack of government support including training [37]. Furthermore, the COVID-19 pandemic has further exacerbated indigenous women’s limited access to public health services due to the closure of services and limited information resulting in fear of contagion [38].

Beyond the specific challenges of structural inequalities and discrimination against Indigenous peoples in Peru, our results point to the challenges inherent in the existing system of support for all women experiencing violence. As previously described, there are important problems in the implementation of the laws due to lack of funding and lack of training. Broader system problems, such as inadequate sanctions for perpetrators and safeguarding of survivors [36, 39] contribute to the perception that reporting is not useful or safe, deterring survivors from reporting. This is consistent with other studies which highlight women’s difficulties reporting violence to police officers, and describe police responses as unsympathetic, impersonal, or indiscreet, as well as women experiencing significant delays in the overall legal process and issuance of protection measures [40].

To address the extent of this problem, systematic changes are needed at multiple intersecting levels.

The health system can play an important role in responding to IPV including identification, supportive response to disclosure, and the provision of clinical care, follow-up, referral, and holistic support for women [41–43]. While inadequate resources and overstretched health services are characteristic of many low- and middle-income countries, low-cost and effective interventions for increasing the reporting of violence and its consequences have been developed in other similar settings [44]. To address the unique needs of indigenous communities, health service provision needs to include linguistic and cultural training for healthcare staff, strategies to address economic barriers to access (e.g., outreach programmes that travel to indigenous communities), and consideration of local approaches to judicial procedures and retribution within a women’s rights framework [45]. Implementation and uptake of legal and policy frameworks for addressing IPV is also needed, as are unique considerations of how these policies can be used to better address the specific needs of indigenous women. Suggestions on how this might be done can be drawn from other settler contexts, such as Australia, where high rates of violence against Indigenous women have been tackled using a holistic policy response which focuses on healing and empowerment, and strong community involvement [46] in policy development [47]. Policy-backed monitoring and evaluation of these changes will provide crucial evidence of their impact.

Drawing on existing scholarship and our findings of the barriers to service access, we support the relevance of the UN Women framework for indigenous communities in Peru. The principles and common characteristics of the framework provide an important focus for understanding service provision for indigenous communities. Of particular relevance for indigenous communities are the principles of a rights-based approach and culturally appropriate and gender-sensitive services. In terms of common characteristics, accessibility, adaptability, acceptability, and appropriateness need to be prioritised by researchers and practitioners. We propose key recommendations for improving service provision for indigenous communities centred around these principles and characteristics: a) outreach of health services to remote communities, with local participation from communities themselves; b) service provision in local languages; c) clear protocols and respect for autonomy within local systems of governance and their integration with national judicial responses to VAWG; d) meaningful involvement of indigenous communities in developing relevant policies; e) intercultural competency training for service providers to build more inclusive and non-discriminatory services; and f) allocation of funds to enable women to access services, including subsidised transportation and child care.

The findings of this study also have implications for research. Our study further confirms the need for research on VAWG and its prevention to involve formal commitment and concrete actions and accountability of local authorities, stakeholders, and the community. Research that includes the evaluation of interventions should ensure that there are strong links to existing services or the creation of appropriate services, which consider the unique needs of indigenous communities. This may involve a considerable amount of preliminary qualitative research to fully understand the enablers and barriers to health and social service access, help-seeking behaviour for women who experience violence, and local community-based mechanisms of support and/or prevention.

### Limitations and strengths

Despite the guarantee of anonymity, some participants were reluctant to participate because they felt unable to represent the institutions that they worked for on the issue of VAWG. Similarly, some participants may have had difficulties speaking freely about their work and related experiences. Some aspects of the UN Women framework were not covered by our interview questions. However, we believe that these are not genuine gaps in service provision but were instead outside of the scope of the interview discussion or the participants’ knowledge. Another important limitation was the lack of face-to-face interviews that could have impacted the answers of participants. Additionally, internet connection was a problem in some interviews given the remote location and lack of infrastructure to support virtual interviews. This might have affected the fluidity of the interview and ability to build rapport with the KIs.

The main strength of our study was the involvement of KIs from different sectors, which enabled us to conduct a multisectoral assessment of the responses and services related to IPV in Amantaní. To our knowledge, this is the first study of IPV services for women from Amantaní or any other islands in the Andes. This work is helpful for further research in this region as whilst focussing on intimate partner violence, it serves to highlight the crucial additional dimension of structural violence. VAWG prevention research would benefit from using a structural violence framework more widely as individual experiences of violence are complexly shaped by structural drivers; women with intersecting marginalised identities, including the indigenous women of Amantaní, experience multiple systemic inequities.

As an example of what a structural violence framing might look like for VAWG, as part of the EVE Project, we have taken these findings on board in assessing the potential implications of conducting research with an indigenous community about violence. As a team, we determined that our original plan of completing a survey across the island to assess VAWG prevalence could potentially put women’s lives in danger given the lack of support services available. We considered the possibility of developing our own support services as part of the project but felt that the risks that women would perceive these supports as external to the community (and therefore potentially stigmatising of their experiences) was still larger than the potential benefits of a survey to the community itself. We therefore redesigned the EVE Project study in Peru around a series of strengths-based workshops to identify local sociocultural assets that could be used to address the issues the community themselves identified as something that needed to be addressed, including IPV.

Moreover, our study is a clear example of how using a multi-disciplinary approach provides a deeper understanding of VAWG, especially in remote indigenous communities. A multi-disciplinary approach is needed to address the intersections between individual experiences of IPV and structural violence, and responding to and preventing violence needs to involve actors beyond the health sector. The lessons learned from indigenous communities emphasise the need for such a multi-disciplinary and holistic approach and could provide leverage in the move towards decentralisation and improving access to current services. Further research is needed to understand how to improve services based on the needs and structural constraints of indigenous populations and valuable insights would be gained by working in meaningful partnerships with indigenous women and people who have lived experiences of violence in this region to advance this agenda.

## Conclusions

IPV is a serious health and human rights issue affecting women in Amantaní who have been traditionally excluded and discriminated against. Service provision in this region needs to adopt a decentralised, intercultural, rights based and gendered approach to IPV response and prevention, considering cultural and linguistic relevance for indigenous populations. The role of structural violence in perpetuating indigenous women’s experiences of violence and undermining their access to services must be central when designing and implementing appropriate policies and services if they intend to meet the needs of indigenous women in Peru.

## Data Availability

Data are available on request.
